# Costs of Treating Uncomplicated Sickle Cell Complications in a Day Hospital Setting in Equatorial Africa

**DOI:** 10.1155/anem/5415432

**Published:** 2025-12-30

**Authors:** Lydie Ocini Ngolet, James Taylor, Amour de Chabervy Atipo, Clement Pach Mikia, Chelsea Bango, Fresnel Samba Bantsimba, Irene Ondzotto Ibatta, Alexis Elira Dokekias

**Affiliations:** ^1^ Department of Hematology, Brazzaville Teaching Hospital, Brazzaville, Congo; ^2^ Marien Ngouabi University, Brazzaville, Congo, umng.cg; ^3^ Division of General Internal Medicine, Virginia Commonwealth University, Richmond, Virginia, USA, vcu.edu; ^4^ Center for Sickle Cell Disease, Departments of Medicine (Hematology and Oncology) and Microbiology and Immunology, Howard University College of Medicine, Washington, District of Columbia, USA, howard.edu; ^5^ Department of Food and Human Nutrition Sciences, University of Manitoba, Winnipeg, Manitoba, Canada, umanitoba.ca; ^6^ National Referral Sickle Cell Center (Centre National de Reference de la Drépanocytose), Brazzaville, Congo

## Abstract

Sickle cell disease (SCD) is a major public health concern in Africa. SCD healthcare expenses are covered by personal finances in the Congo due to the absence of universal public health insurance. Various strategies have been implemented to minimize this economic burden, including an outpatient management strategy. The present study evaluated the costs associated with outpatient management of uncomplicated sickle cell crises. Patients presenting to the National Sickle Cell Center’s emergency room between December 2023 and February 2024 were included in this study for a total of 114 subjects with uncomplicated crises from SCD. Chief complaint, diagnosis, treatment, health insurance coverage, and cost of care were recorded for each subject. We further assessed the consistency of treatment with published standards of care. Mean patient age was 16 ± 13 years, where more than one‐third were adults (36.5%). The ratio of males to females was 1.09. Only 26.2% were employed. Monthly income was lower in individuals with SCD ($310.32 ± 120.93) compared to those without SCD ($386.92 ± 471). Managing uncomplicated SCD as an outpatient costs an average of $99.86 ± 49, where medications represented 55.7% of the total expense. Sixty‐six patients (57%) received prescription medications and investigation with no rational basis, resulting in an inflated cost of $15.96 per person. The present study did not demonstrate any financial benefits to managing uncomplicated SCD in outpatient settings.

## 1. Introduction

Sickle cell disease (SCD) is an inherited hemoglobinopathy and the most prevalent monogenic disorder affecting populations worldwide, with most in Sub‐Saharan Africa. It ranks as the 12th leading cause of death among children under five years old [1]. SCD is associated with significant morbidity resulting in frequent healthcare visits for both outpatient care and inpatient hospitalization. In the United States., the cost of managing acute SCD pain in an inpatient setting is estimated at $17,537 annually [2], while day hospital care costs are significantly lower at $6891 [2]. In the United States, individuals with SCD and private insurance have a total lifetime cost of $1.7 million for SCD‐related medical expenses, of which $44,000 are out‐of‐pocket costs [3].

In Sub‐Saharan Africa, there were 405,000 live births with SCD in 2021 [1]. 90 percent of children with SCD die due to late diagnoses, lack of access to proper treatment, and treatment costs exceeding affordability. Survivors are at an increased risk of infections, vaso‐occlusive crisis, severe anemia, stroke, and other morbidities that require costly preventive and curative interventions. Across Africa, insurance coverage is low or nonexistent, and therefore, medical expenses are borne by families. Consequently, disparities in access to care and service quality are observed depending on the socioeconomic status of the patient or their family. The Republic of Congo is a country located in the equatorial region of the African subcontinent with an approximate population of 5 million and 75,000 SCD patients (estimated 1.5% of the population). The country has one of the highest sickle cell trait prevalence in the world and has not yet established a neonatal SCD screening program [4]. SCD mortality rate remains high, 36% for children younger than age 5 years and 43.3% for those aged under age than 10 years [5]. No access to diagnosis tools, poor management, denial of the disease by families, and their low socioeconomic status are among the factors that may explain the burden of the hemoglobinopathy [6].

To reduce these inequalities, multiple strategies have been implemented in Congo. One strategy was the creation of a National Comprehensive Sickle Cell Center located in Brazzaville. Brazzaville is the capital and largest city of the Republic of Congo, where over 2.1 million people live, which is more than one‐third of the country’s total population. The prevalence of the major type of sickle cell is 1.35% and 19.43% for the sickle cell trait [7]. The center offers a platform for SCD training, research, and care. Sickle cell crises are cured promptly and appropriately, thereby reducing hospitalization time. The sickle cell center manages uncomplicated sickle cell crises in a day hospital setting. The day hospital model reduces the cost of care while offering high‐quality services and allows patients to remain in their familiar environment. The cost of SCD crisis management in inpatient settings is known in the Congo [8, 9]. The monetary value of outpatient day hospital management has not been quantified yet. Here, we estimate the cost of care provided in a day hospital of the National Sickle Cell Center in Brazzaville, which is the only health facility dedicated to sickle cell care in the country. We hypothesized that day hospital would incur lower cost compared to SCD crises treatment in inpatient settings. Such cost‐based analyses will aid the evaluation of our disease management strategies and provide data to the Ministry of Health and other partners/stakeholders.

## 2. Patients and Methods

### 2.1. Description of the Facility

The National Sickle Cell Center (Center National de Reference de la Drépanocytose or CNRD) is located within Brazzaville teaching hospital’s compound in a two‐storey floor building. The first floor includes a waiting area, reception, triage room, day hospital beds, a laboratory, imaging department, physiotherapy, pharmacy, meeting/teaching area, and consultation rooms. Administrative and executive offices, as well as conference rooms, are located on the second floor. The center was established in 2015 by decree 28‐2015 and operates from Monday to Sunday, 8:00 a.m. to 6:00 p.m. The center aims to provide high‐quality care to the SCD community and employs 3 hematologists, 2 pediatricians, 4 residents, 2 radiologists, 1 laboratory medicine specialist, 1 assistant, and 15 nurses. It is a public healthcare facility with a nonprofit policy. Fees for services, interventions, and medicines are among the lowest in the country.

The day hospital unit receives patients from the triage area who are experiencing uncomplicated sickle cell crises requiring parenteral treatment. Uncomplicated sickle cell crises refer to painful crises or anemic crises without acute chest syndrome, stroke, priapism, severe infection (temperature above 38.5 Celsius), and evidence of organ failure or life‐threatening complications. Painful crisis is managed with hydration and analgesics (nonsteroid anti‐inflammatory, codeine, or morphine). Transfusion of packed red blood cells is indicated when the hemoglobin rate drops below 6 g/dL or when the patient develops symptoms related to anemia. Individuals with complicated, exacerbated, or prolonged sickle crises are referred to the hematology department of the teaching hospital for inpatient admission. In the day hospital area, a physician or resident assesses patients. Investigations (imaging and/or lab studies) and treatment plan are daily documented on a day hospital sheet.

The day hospital documentation sheet is divided into four parts:

- Sociodemographic: Full name, date of birth, address, profession, schooling, health insurance, and funding source for medical expenses.
- Clinical: Date, purpose of the visit, vital signs, diagnosis, and evolution/outcome.
- Treatment: Medications, route of administration, dosage, and blood transfusion.
- Investigation: Imaging and blood work listed.

The treatment part has three columns, with each column representing one day of treatment. Subjective, objective, and diagnostic information are documented on a chronological sheet, which is filed in the patient’s chart. The patient is discharged when clinical signs have sufficiently resolved, and further observation or intervention is no longer needed. Outpatient care for an uncomplicated SCD crisis is limited to a maximum of 3 days.

### 2.2. Methodology

This was a descriptive study conducted at the sickle cell center in Brazzaville for 90 days, inclusive of the period between December 15, 2023, and February 14, 2024.

Data collected for analysis included sociodemographic, clinical, diagnosis, treatment, and evolution/outcome characteristics for patients treated for uncomplicated sickle cell crisis during the study period. To avoid loss of information, we collected the day hospital treatment sheet daily. The total number of outpatient visits to the day hospital was based on the number of treatment columns (days) with completed data entry. The direct total cost of managing uncomplicated sickle cell crises in the day hospital was calculated using the cost of each medicine, the total number of red cell transfusion units administered, lab tests, imaging, and the day hospital fee (XAF 5000/USD 8.18 daily). Expenditures were initially calculated in XAF (Central African currency) and then converted to USD at a conversion rate of 1 USD = 611.04 XAF. Some ancillary costs were not included in the cost estimate including intravenous kits or indirect costs. The cost of blood transfusion covers the pretransfusion blood type and the blood products in the Congo. There is no hemovigilance unit or pre‐ and posttransfusion screening program in place. Therefore, serology testing before and after any blood transfusion is not a part of the protocol. Furthermore, our laboratories do not have the capacity to screen for irregular agglutinins.

Overtreatment is prevalent in sickle cell patients and contributes to escalating healthcare costs. In this study, we have conducted a second analysis to quantify its financial impact. We assessed the appropriateness of managing uncomplicated sickle cell crisis according to our local guidelines, which are aligned with the WHO package of interventions for SCD. Interventions, including both drug prescription and diagnosis investigation, were labeled rational when they adhered to our guidelines and irrational when they did not. We then calculated the costs attributable to irrational management costs by summing the costs incurred by each intervention.

We did not include the intercritical care cost, which comprises vaccines, folic acid, antibiotic prophylaxis, and hydroxyurea.

### 2.3. Ethical Considerations

The ethical approval for the study was obtained from the CERSSA, Ethic Committee for Health Sciences Research in Brazzaville, Congo (Approval ID: N°223MESRIT/DGRST/CERSSA/‐24). Written consent was provided by each participant aged 16 years or older. All participants were able to provide written consent. For participants under the age of 16, informed consent was obtained from parents or legal guardians, each of whom was able to provide written consent.

### 2.4. Statistical Analysis

Data were recorded using Microsoft Excel (Microsoft Corporation, Redmond, WA) and analyzed with statistical software including Prism (Graph Pad Software). Quantitative variables were described using percentages, means, and standard deviations. The effects of sociodemographic, clinical, treatment, and investigation variables on treatment cost were analyzed using ANOVA and Student′s *t*‐tests, with significance set at *p* < 0.05.

## 3. Results

### 3.1. Patient Demographics

During the 90‐day study period, 487 subjects with SCD received care at the sickle cell center. Of these, 299 (61.4%) were scheduled consultations, and 188 (38.5%) presented as unscheduled walk‐in visits. Among unscheduled walk‐in visits, 73 patients were diagnosed with complicated sickle cell crises and referred to the hematology department, while 115 (61.2%) were admitted for day hospital care as previously described. Sixty cases of these admissions were male and 55 were female, yielding a sex ratio of 1.09.

Treated patients ranged in age from 2 to 73 years, with a mean age of 16 ± 13 years. The largest demographic group were children 6–17 years (*n* = 57; 63.5%). Adults accounted for 42 individuals (36.5%), among whom, 11 (26.2%) were employed, 2 (4.8%) unemployed, 1 (2.3%) retired, and 28 (66.7%) were pursuing school or college.

None of the patients had health insurance. Medical expenses were entirely covered by parents (100%) for children and adults pursuing school or college. For adults, not enrolled in school or college, 92.9% (*n* = 13) self‐funded care, while 7.1% (*n* = 1) relied on financial support from relatives or extended families. The mean monthly income of employed adults with SCD was 189,615 XAF ± 73,894 ($310.32 USD ± 120.93). The mean income of parents and relatives/extended families of patients with SCD was 236,419 XAF ± 287,906 ($386.92 USD ± 471.18). The main diagnoses warranting day hospital admission were anemic crises (*n* = 52; 45%), pain crises (*n* = 48; 42%), and mixed crises involving anemic and pain crises (*n* = 6; 5.2%). These episodes were predominantly associated with clinical evidence of infections (97.4%, Table 1).

**Table 1:** Sociodemographic characteristics.

Characteristics	*N* (%)
Gender	
Male	60 (52.2)
Range of age (years)	
0–17	73 (63.5)
18–39	33 (28.7)
40 and over	9 (7.8)
Patients’ employment status	
Unemployed	2 (1.7)
Retired	1 (0.9)
Employee	11 (9.6)
Schooling	101 (87.9)
Funding source of medical expenses	
Relative/extended family	1 (7.1)
Self	13 (92.9)
Parents	100 (87.0)
Day hospital diagnosis/justification	
Anemic crisis	52 (45.0)
Pain crisis	48 (42.0)
Mix crisis	6 (5.2)
Causal factor of crisis	
Acute respiratory infection	48 (42.0)
Malaria	46 (40.0)
Diarrhea	7 (6.1)
Osteomyelitis	3 (2.6)
Hepatobiliary infection	3 (2.6)
Undetermined infection	3 (2.6)
Leg ulcer	3 (2.6)
Stress	3 (2.6)

### 3.2. Medical Expenses Related to the Sickle Cell Center Day Hospital

The duration of SCD day hospital care averaged 2.6 ± 0.6 days. The average medical expenses for treatment of uncomplicated SCD crises ranged from XAF 22,400 (USD $36.66) to XAF 153,400 (USD $251.05), with a mean total cost of XAF 61,019 (USD $99.86).

Medication costs constituted the largest proportion of the total expenses accounting for 55.7%, followed by day hospital fees at 22.2% and blood transfusions at 20.9%. Laboratory tests and imaging services were the least costly components, comprising only 16.4% of the total expenditure. The cost of care varied significantly by patient age group, with the highest expenses observed among individuals aged 18–39 years (*p* = 0.037, Table 2).

**Table 2:** Cost components of SCD day hospital management.

Age groups (years)	0–17 (*n* = 73)	18–39 (*n* = 33)	40 (*n* = 9)	All (*n* = 115)	*P*
Day hospital LOS	2.77 (0.51)	2.70 (0.77)	2.33 (0.87)	2.6 (0.63)	0.13
Day hospital fees^∗^	13,836/22.64 (2571/4.21)	13,485/22.07 (3850/6.30)	11,667/19.09 (4330/7.09)	13,565/22.20 (3160/5.17)	0.13
Drug cost^∗^	33,243/54.40 (17,158/28.08)	39,882/65.27 (23,177/37.93)	17,756/29.06 (7541/12.34)	33, 936/55.54 (19,736/32.30)	0.005
Investigation fees^∗^	10,114/16.55 (4667/7.64)	10,185/16.67 (4764/7.64)	7800/12.77 (2049/3.35)	10,020/16.40 (4599/7.53)	0.5
Blood transfusion fees	10,909/17.85 (3822/6.25)	14,464/23.67 (2004/3.28)	15,000/24.55 (0/0)	12.733/20.84 (3485/5.70)	0.002
Total expenses	59,860/97.97 (21,800/35.68)	67,836/111.02 (27,511/45.02)	45,422/74.34 (19,591/32.06)	61,069/99.94 (29,938/49)	0.037

^∗^Mean in XAF/USD (DS: XAF/USD).

### 3.3. Evaluation of Care

Fifty‐seven patients (57%) received prescriptions without justification, classified as irrational. This primarily consisted of irrational drug prescriptions (43%), the majority of which were antimalarials (69.5%). Forty‐two patients (36.5%) underwent investigations deemed irrational. Table 3.

**Table 3:** Features of irrational prescriptions.

Irrational management	*N* (%)
Antimalarial	41 (69.5%)
Antibiotics	18 (30.5%)
Irrational investigation	42 (36.5%)
Malaria smear	35 (83.3%)
X‐Ray	7 (16.7%)

The average cost associated with the irrational management of uncomplicated SCD was XAF 9750 (USD $15.96) per patient, with expenses ranging from XAF 1000 (USD $1.63) to XAF 52,000 (USD $84.92).

## 4. Discussion

The cost of healthcare in Sub‐Saharan Africa imposes a significant financial burden on individuals with SCD and their families. In the absence of private insurance, universal health coverage, or government subsidies, out‐of‐pocket payments remain the primary means of accessing the only financial means of accessing healthcare. For chronic diseases such as SCD, which require ongoing medical management, high out‐of‐pocket expenses can drive families into poverty, cause social strain, contribute to patient discrimination, and, in severe cases, lead to child abandonment. Therefore, understanding the true costs of SCD care is essential to appreciating the disproportionate economic burden of this disease. Accessories, pointing out the cost of irrational care, will help to review guidelines and strategies. Such data can also inform advocacy, national policy development, financial assistance programs, and critical evaluations of care delivery strategies.

We have quantified the cost of managing uncomplicated sickle cell crises in a day hospital setting at the National Sickle Cell Center in Brazzaville, which is one of two major cities where one‐third of the Congolese population lives. Previous studies in 2016 highlighted the financial hardship experienced by families of children and adults with SCD admitted to the intensive care unit and hematology department [8, 9]. To respond to patient needs, a National Sickle Cell Center was created in Brazzaville as an initial step toward an SCD national policy [4]. The center’s mission is to improve the lives of individuals affected by SCD by providing high‐quality care, conducting research, empowering the SCD community, and raising public awareness. The center is an outpatient facility where uncomplicated SCD crises can be treated in a day hospital setting instead of an inpatient hospital ward. The day hospital was implemented to personalize care, reduce disruption of patient lives, and lower the cost of SCD treatment.

We found that treating uncomplicated SCD manifestations in a day hospital costs an average of 61,019 XAF ($99.94) per patient, with a wide range from 22,400 XAF ($36.66) to 153,400 XAF ($250.05). These figures are consistent with prior studies in Africa, which have reported costs ranging from $33.9 to $229 [10–12]. In contrast, earlier estimates in Brazzaville placed the cost of SCD crisis management in the intensive care and hematology department at 99,889 XAF ($163.08) and 103,110 XAF ($163.08–168.39). In both settings, the cost of care remains too high for the average family, which often results in heightened financial vulnerability [13, 14]. Indeed, recurrent emergency visits and hospitalizations contribute to work absenteeism and increased risk of employment loss [15, 16]. Consequently, adults with SCD have lower incomes than their peers as illustrated by the present study: 189,615 XAF ($308.92) versus 236,419 XAF ($386.09).

Children with SCD experience significant academic challenges, including neurocognitive and psychological difficulty as well as higher school absenteeism, leading to disruption of academic promotion [17–20]. Consequently, individuals remain at school/college into adulthood. In our study, roughly one‐third of adult patients were still pursuing education.

Day hospital fees represented a higher proportion of total medical expenses (22%) than inpatient fees (10% of total charges). Contrary to our hypothesis, day hospital care did not emerge as a less costly delivery model for SCD management. Low‐income SCD patients preferred admission to the teaching hospital where a flat hospitalization fee of 15,000 XAF ($24.47) is charged regardless of the length of stay [8, 9]. In addition, a further discount of 5000 XAF ($8.1) is usually granted to those who cannot afford the full amount of hospitalization. By comparison, the day hospital charges 5000 XAF ($8.1) per day, resulting in similar overall costs for typical three‐day outpatient management of SCD crises without any discount. From the patient’s perspective, inpatient care may appear more financially predictable.

When considering only the cost of care (excluding hospital fees), the day hospital cost was approximately 21.9% less expensive than full hospitalization averaging about 30,000 XAF ($49.10). However, this comparison is limited by several factors: care in intensive and hematology units involves more severe cases, requiring longer treatment duration, admission (2–7 days), and more resources. When comparing the pediatric population in both settings, the outpatient savings narrowed to only 7.3%. These findings suggest that the perceived cost advantage of day hospital care over inpatient care is modest, highlighting the need for more detailed cost analyses.

Blood transfusion was the second most expensive component in day hospital expenses, whereas it ranked third in other studies [11, 12, 21, 22]. All the patients transfused experienced deep anemia symptoms; therefore the indication of transfusion was correct. However, as reported by Cansian et al. in Brazil, the quantity of blood prescribed should be reconsidered. Analyses have shown that the volume of red blood cells prescribed was inadequate in 17.5% [23]. Children represented 63.5% of our study population. All of them received adult red cell packs, suggesting that the quantity of blood prescribed and transfused was not appropriate. Blood‐related expenses were similar in the pediatric and adult populations. The Congo, like many countries in the region, decentralized blood production and limited government subsidies and disincentivized blood production services [24, 25]. They lead to constrained supplies and suboptimal practices, including the use of adult packed red cells in children. This approach not only increases costs but also contravenes WHO recommendations for pediatric transfusion. Given the high rate of anemic crises requiring transfusion, interventions such as hydroxyurea therapy offer opportunities to reduce transfusion rates, and hence the cost of care [26].

Drug costs accounted for 55% of the total expenses in the day hospital, consistent with the observations by other authors [10, 12]. Fifty‐seven percent of patients received prescriptions that were inconsistent with WHO and our guidelines for SCD management, involving unnecessary antimalarial treatment [27–30]. Irrational medication prescription costs resulted in an average excess cost of 9000 XAF ($14.70) extra per patient. The use of standardized treatment algorithms for uncomplicated SCD crises aimed at reducing the variability between physicians could have resulted in significant savings in the day hospital (52,019 XAF, $85.34). We speculate that there are opportunities for identifying further savings in the outpatient day hospital setting, but this will require prospective analysis of other factors including length of treatment, medication dosage, and route of medication administration. Finally, there are some important limitations for our analysis. We have not addressed additional costs associated with uncomplicated SCD crisis including transportation, cafeteria services, and perceptions of caregivers. Future studies will be required to replicate our findings and incorporate these additional factors into total costs. A comprehensive understanding of cost will allow for future modification of our day hospital strategy such that it is able to achieve its intended goal of lower cost, high quality, and uniform availability to patients.

## 5. Conclusions

An uncomplicated SCD crisis can be effectively managed in outpatient settings. It requires a well‐trained workforce and reinforcement of standardized clinical guidelines to ensure rational treatment.

## Conflicts of Interest

The authors declare no conflicts of interest.

## Author Contributions

Irene Ondzotto Ibatta, Clement Pach Mikia Alexis and Elira Dokekias contributed to the study, Lydie Ocini Ngolet designed the study. Amour de Chabervy Atipo and Fresnel Samba Bantsimba collected the data. Chelsea Bango analyzed the data and supervised the research. Lydie Ocini Ngolet and James Taylor wrote and reviewed the manuscript.

## Funding

The research did not receive any funding.

## Data Availability

The data that support the findings of this study are available on request from the corresponding author.
